# Development of Green and Facile Sample Preparation Method for Determination of Seven Neonicotinoids in Fresh Vegetables, and Dissipation and Risk Assessment of Imidacloprid and Dinotefuran

**DOI:** 10.3390/foods13071106

**Published:** 2024-04-04

**Authors:** Osama I. Abdallah, Rania M. Abd El-Hamid, Nevein S. Ahmed, Saleh S. Alhewairini, Sherif B. Abdel Ghani

**Affiliations:** 1 Department of Pesticide Residues and Environmental Pollution, Central Agricultural Pesticide Laboratory, Agricultural Research Center, Giza 12618, Egypt; ranianana82@yahoo.com (R.M.A.E.-H.); dr.nevein@gmail.com (N.S.A.); 2 Department of Plant Protection, College of Agriculture and Food, Qassim University, P.O. Box 6622, Buraydah 51452, Saudi Arabia; hoierieny@qu.edu.sa; 3 Department of Plant Protection, Faculty of Agriculture, Ain Shams University, P.O. Box 68 Hadayek Shoubra, Cairo 11241, Egypt

**Keywords:** green solvent, neonicotinoid pesticides, residue dissipation, method validation, risk assessment

## Abstract

A facile procedure for extracting and determining seven neonicotinoids was developed. Water was the only extraction solvent without phase separation and cleanup steps. The method was validated according to European Union standards, and the values obtained were compared with the criteria. The accuracy values were between 99.8% (thiamethoxam) and 106.8% (clothianidin) at the spiking levels of 0.01, 0.1, and 1 mg/kg in the tested matrices. The precision as pooled RSD values was ≤6.1% (intra-day) and ≤6.9% (inter-day). The limit of quantification was set and tested at 0.01 mg/kg. The matrix effect was evaluated, and all matrices had a suppressive effect. The matrix of the cucumber was the most effective, with −20.9% for dinotefuran and an average of −9.8% for all compounds, while the tomato matrix had the slightest effect. Real marketed samples were analyzed using the developed and QuEChERS (Quick, Easy, Cheap, Effective, Rugged, and Safe) methods; the results were not significantly different. A supervised field trial was conducted in the open field to study the dissipation patterns of imidacloprid and dinotefuran in tomatoes. The dissipation of both compounds followed first-order kinetics. The half-life (T_½_) values were 3.4 and 2.5 days, with dissipation rates k of 0.2013 and 0.2781 days, respectively. Following the EU-MRL database, the calculated pre-harvest interval (PHI) values were 7 and 14 days for imidacloprid and dinotefuran, respectively, and 3 days for both compounds following Codex Alimentarius regulations. The risk of imidacloprid and dinotefuran residues was estimated from chronic and acute perspectives. The risk factors of dinotefuran were lower than those of imidacloprid. Nonetheless, the highest expected residues of both compounds were below the tolerance limits.

## 1. Introduction

Neonicotinoids are a class of pesticides analogous to nicotine. They are neurotoxic compounds that act as agonists of nicotinic acetylcholine receptors (nAChRs) [1]. Unlike nicotine, neonicotinoids have a higher affinity towards insects’ nAChRs than mammals, resulting in high selectivity for insects while being safer for mammals [2,3]. Neonicotinoids are systemic insecticides with high water solubility.

Neonicotinoids are consumed in more than half of the world’s countries [4], with a share of about 25% of insecticide usage [5]. Neonicotinoids, like other plant protection chemicals, are used deliberately to control pests, and their residues might pose risks to consumers [6], wildlife [7], honeybees [8], etc. Agricultural products, by law, are monitored for pesticide residue content. The need to overcome problems of older methods fuels the search for newer ones to improve sensitivity and specificity, decrease cost, and use safer solvents or decrease their amounts.

Most data on neonicotinoid residues in fruits and vegetables were from brief reports on applying the newly developed analytical methods. Neonicotinoids were the subject of multi-residue analysis methods among other compounds [9,10]. Otherwise, they were targeted by specialized methods only tailored for multi-neonicotinoids [11,12,13,14,15] or a single compound, such as clothianidin [16], thiacloprid [17], and sulfoxaflor [18]. The methods mentioned earlier mainly used QuEChERS, modified QuEChERS, or QuEChERS, followed by DLLME (dispersive liquid–liquid microextraction). Various matrices were studied, such as environmental samples [12,19], animal tissues [9,10,13,15], and fresh crops [14,16,17,18].

The liquid–liquid extraction (LLE) of matrices with high water content involves two steps: the first step is the extraction step, in which the sample is blended or mixed with a water-miscible solvent. The second one is a cleanup step wherein a water-immiscible solvent, depending on the polarity of the targeted analytes, is added to the extract, and the extractants are partitioned between them [20]. In the QuEChERS method, the first step is the same as in LLE: a water-miscible solvent is mixed with the sample puree, while the second step is an in situ cleanup step achieved by the addition of salt mixtures to partition the extractants between the aqueous salt phase and the extracting solvent [21]. The sample solvent ratio is 1:1 [21] or reaches 1:3 [20], resulting in a high content of co-extracted materials that may require an extra cleanup step using a column, SPE, or DSPME to remove them.

Analytical chemists with an eco-friendly focus are currently exploring possibilities for “green” analytical methods to replace polluting ones with cleaner alternatives. They strive to eliminate hazardous chemicals and develop environmentally conscious methods, maintaining high analytical performance. Sample preparation is an essential step in the analytical process for the purposes of the separation and enrichment of target analytes, removal or minimizing matrix interferences, and ensuring instrument compatibility. On the other hand, sample preparation requires solvents (solvent extraction techniques), sorbents (solid-phase extractions), reagents (for derivatization reactions or the removal of impurities), acids or bases (for pH correction or mineralization), energy input (heating, stirring, cooling), and other consumables or equipment (such as cartridges and pipette filter tips), which should be used in a very environmentally conscious manner [22].

Therefore, this study aimed to develop and validate a facile, environmentally friendly, and analyst-friendly method for the determination of seven neonicotinoids in fresh and high water-content vegetables in a dilute and shoot fashion using water only for extraction. The developed AGREE metric approach [23] was used to evaluate the environmental greenness of the proposed analytical method. To our knowledge, this is the first time that such a study has been conducted on neonicotinoids. The development parameters and validation criteria were studied, and method applicability was given attention by determining the actual samples collected from the local markets and comparing the results with the standard EN QuEChERS method [24]. In addition, we investigated the dissipation patterns and estimated the pre-harvest intervals (PHIs) of two representatives of neonicotinoids, imidacloprid and dinotefuran, in tomatoes grown under open field conditions. Finally, a risk assessment was carried out for the residues resulting from the field trial based on the calculation of acute and chronic risk factors.

## 2. Materials and Methods

### 2.1. Chemicals

All analytical standards of neonicotinoids (≥99.9%) were purchased from Sigma-Aldrich (St. Louis, MO, USA); formic acid, glacial acetic acid, and ammonium formate (LC-MS grade) were obtained from Sigma-Aldrich (St. Louis, MO, USA). Acetonitrile (pesticide residue grade) was purchased from Fisher Scientific (Loughborough, UK). QuEChERS EN extraction kits (4 g MgSO_4_, 1 g NaCl, 1 g trisodium citrate dihydrate, and 0.5 g disodium hydrogen citrate sesquihydrate) and primary secondary amine bulk sorbent (PSA) were purchased from Agilent Technologies Inc. (Wilmington, DE, USA). Anhydrous magnesium sulfate (MgSO_4_) (purity, >98%) was purchased from Chem-Lab NV (Zedelgem, Belgium). Commercial formulations of imidacloprid and dinotefuran were bought from local markets.

### 2.2. Standard Solutions

Stock standard individual solutions of 1 mg/mL (dinotefuran, nitenpyram, thiamethoxam, clothianidin, imidacloprid, acetamiprid, and thiacloprid) were prepared in acetonitrile. A working standard mixture solution (WMS) of 1 mg/L of each analyte was prepared freshly by diluting stock solutions in deionized water and used for further dilutions. The dilutions used for a standard calibration curve in solvent (SS), i.e., 0.0005, 0.001, 0.0025, 0.005, 0.01, 0.025, 0.05, 0.1, and 0.25 mg/L for each analyte, were prepared in 30 mL water in Falcon tubes. As in the case of SS, matrix-matched standard (MMS) calibration mixture solutions were made using the corresponding extract of blank samples in place of water. MMS was utilized in calculations of the matrix effect and residue determination for field and market samples. Stock solutions were stored at −20 °C; SS, WS, and MMS were stored at +4 °C.

### 2.3. Sample Preparation

A concise extraction process without a cleanup step was utilized. Deionized water (about 27 mL) was added to three grams of a thawed analytical sample or blank sample (±0.03 g) in a 50 mL Falcon tube to make it up to 30 mL. Tubes were shaken by hand for 1 min. Then, 1 mL of the solution was filtered through a 0.22 micron sample filter into an injection screw Teflon-capped vial. Vials were subjected to LC-MS/MS analysis. The standard EN QuEChERS method [24] was used to validate the results of the proposed method.

### 2.4. LC-MS/MS

A Dionex Ultimate 3000 RS UHPLC + focused system coupled with a TSQ Altis triple quadrupole mass spectrometer (MS/MS, Thermo Fisher Scientific, Austin, TX, USA) was used to detect residues of the seven neonicotinoids. Trace Finder software (version 4.1) was used for analysis, data acquisition, and reporting. An Accucore RP-MS C18 column (150 × 2.1 mm, 2.6 μm film thickness, Thermo Fisher Scientific) was used for separation at 40 °C. Mobile phase A was water, and mobile phase B was methanol; the flow rate was 0.30 mL/min. The gradient program of the mobile phase was 0–2 min 10% B, 2–6 min from 10% B to 90% B, 6–8 min 90% B, 8–8.1 min from 90% B to 10% B, and 8.1–16 min 10% B. The injection volume was 2 μL. To optimize the MS/MS parameters of the tested analytes (Table S1), a Harvard infusion pump (Harvard Apparatus, South Natick, MA, USA) was used. The precursor ions [M + H]^+^ were identified in the multiple reaction monitoring (MRM) mode using an electrospray ionization interface in the positive ion mode (H-ESI+). The MS conditions were as follows: the ion source temperature was set at 325 °C, the ion spray voltage was set at 3800 V, and the sheath and auxiliary gasses were 40 and 10 arb, respectively. Trace Finder (version 4.2) software was used for data acquisition.

### 2.5. Method Development and Validation

Method development involves testing different analytical parameters and combinations to determine a particular analyte(s), qualitatively or quantitatively [25]. Tomato, lettuce, and cucumber were selected to represent different matrices and groups of vegetables. Also, tomatoes, cucumbers, and lettuce are targets for many insect pests that frequently require chemical control using neonicotinoids. Different parameters of the method were assessed to attain the best combination for the method. Different injection volumes of 1, 2, 3, 4, and 5 µL were tested. Extraction solvent: different extraction solvent mixtures in water were tested, i.e., 0, 10, 20, and 30% acetonitrile and methanol in water. The different extraction mixtures were analyzed using the obtained absolute areas. Extraction time: handshaking times viz. 1, 2, and 3 min were tested. Centrifugation/filtration: different centrifugation times and forces were tested. Stability of residues in water extract: the stability of residues in injection vials in an auto-sampler at laboratory ambient temperature (22 °C) for 24 h was tested in pure water and water extracts at the 0.1 mg/L level.

Validation parameters were studied: the linearity, accuracy, precision, matrix effect, and LOQ. Linearity, as R^2^, was assessed by composing a matrix-matched standard curve by plotting the average area of three replicates against the corresponding concentration (0.0005, 0.001, 0.0025, 0.005, 0.01, 0.025, 0.05, 0.1, and 0.25 mg/L) using Microsoft Excel. The R^2^ values for tomato, cucumber, and lettuce were obtained from the regression line equation.

The method’s accuracy for each compound was evaluated experimentally by calculating the average recovery percentage. A blank sample of tomato, cucumber, or lettuce (pre-tested to ensure the absence of the target analytes) was weighed in a Falcon tube and spiked with an appropriate volume of standard solution to achieve levels of 0.01, 0.1, and 1 mg/kg (n = 5 replicates for each level). The sample was mixed with a spatula and left to stand for 20 min, then extracted and determined using the proposed procedure. The method’s precision for each analyte was calculated using Equation (1) as the pooled relative standard deviation (pooled RSD) of recovery experiments data at the three spiking levels analyzed on the same day (intra-day repeatability, pooled RSDr). Inter-day repeatability (pooled RSD_R_) was evaluated on different days (three times with 7 day intervals) at only a 0.01 mg/kg spiking level.

(1) $${RSD}_{Pooled}=\sqrt{\left(\frac{\left(n_{1}-1\right){RSD}_{1}^{2}+\left(n_{2}-1\right){RSD}_{2}^{2}+\left(n_{3}-1\right){RSD}_{3}^{2}}{\left(n_{1}-1\right)+(n_{2}-1)(n_{3}-1)}\right)}$$

For the RSDr calculation, n_1_, n_2_, and n_3_ represent the number of replicates, and RSD_1_, RSD_2_, and RSD_3_ represent the relative standard deviations of lettuce, tomato, and cucumber, respectively. For the RSD_R_ calculation, n_1_, n_2_, and n_3_ represent the number of replicates, and RSD_1_, RSD_2_, and RSD_3_ represent the relative standard deviations of the tested matrices on days 1, 2, and 3, respectively.

According to the European Union database, the LOQ was verified at 0.01 mg/kg as the lowest MRL [26]. The matrix effect was calculated using the slopes of the constructed matrix-matched standard and standard in solvent (water) curves using Equation (2) [17].

(2) ME=((slope of MMS)/(slope of SS)−1)×100

### 2.6. Blank and Actual Market Samples

Blank (untreated) tomato, lettuce, and cucumber samples were collected from organic farms that had not recently used the tested neonicotinoids. Samples were collected and treated according to the European Commission guidelines (European Commission Directive (2002/63/EC) [27] and Commission Regulation (EC) No 178/2006) [28]. Samples were analyzed using QuEChERS EN [24] to ensure they were free of the studied compounds. Pesticide-free samples were utilized to prepare matrix-matched standards and in linearity, LOQ, accuracy, and precision studies. Tomatoes, lettuce, and cucumbers (10 samples from each crop) were randomly collected from a local market and analyzed using the standard EN QuEChERS procedure [24]. Then, the samples were re-analyzed using the proposed developed method to confirm and quantify the negative and positive samples.

### 2.7. Field Trial

Field trials were conducted on tomatoes cultivated in open fields, 30.46 N, 30.93 E, Minouf district, Minoufia Governorate, Egypt. The experiments were carried out during January 2022. The average temperature was 15 °C (25 °C as the maximum), relative humidity was 60%, and daylight time was about 10 h during the experiment period. The experimental area was split into plots (25 m^2^ each), and three plots were designated for each pesticide in a complete randomized design. Optirid (imidacloprid 70% Water-Dispersed Granules (WDGs)) at 21 g of active ingredient (a.i) per 100 L spray solution and Tokida (dinotefuran 20% Soluble Granules (SGs) 25 g (a.i.) per 100 L spray solutions were applied using a 20 L knapsack sprayer (purchased from the local market). Full coverage, targeting all foliage, until the run-off of the spray solution was achieved. Samples were collected at 0 (3 h), 1, 3, 7, and 10 days after application. Following the European Commission guidelines (European Commission Directive (2002/63/EC) [27] and Commission Regulation (EC) No 178/2006) [28], 12 primary samples were collected from each plot and combined to make a bulk sample (≥2 kg) of which one kg was taken to the laboratory in a paper bag. Untreated samples were collected before spraying. Samples were comminuted and kept frozen at −20 °C until analyzed. The dissipation rate K was calculated as the additive inverse of the slope of the constructed regression line of the logarithm of residues versus time.

### 2.8. Statistical Analysis

A one-way analysis of variance (ANOVA) and *t*-tests were performed using the data analysis package of Microsoft Excel 2016 to statistically analyze the effect of the parameters of the method and compare residue results of the developed method, respectively.

## 3. Results and Discussion

### 3.1. Method Development and Validation

Injection volume: At 2 µL volume, all compounds’ peaks are typically shaped. Dinotefuran and nitenpyram gave broad or two-tipped peaks at 3, 4, and 5 µL volumes. A 2 µL volume was chosen for injection, and the detection quality was unaffected. Representative chromatograms are shown in Figure S1. Extraction solvent: No significant differences (*p* ≥ 0.05) existed between all tested mixtures at the 0.02 mg/kg level. Additionally, 100% water showed a comparable recovery percent of all compounds. Water was selected as the extraction solvent.

Extraction time: The absolute obtained areas of the tested shaking times showed no significant difference (*p* ≥ 0.05). One minute of handshake was opted to be implemented in the method. Centrifugation/filtration: All forces and times utilized were insufficient to sediment all particulates and needed extra filtration steps. Fortunately, only one mL is required for the chromatograph. Filtration using a sample filter (0.22 microns) was employed, achieving the required result quickly. Stability of residues in water extract: The absolute areas obtained for tested compounds directly after preparation (zero time) and after 24 h of incubation in an auto-sampler were compared. A standard solution in pure acetonitrile (as in the QuEChERS method) was utilized for normalization. A *t*-test statistical analysis showed no significant difference (*p* ≥ 0.05) between compounds’ areas before and after incubation.

It is noteworthy to report that the obtained extract using water was particulate-free and diluted enough to minimize the co-extracted materials without affecting the detection efficiency of the method.

Linearity, matrix effect, and LOQ: Calibration curves were plotted using the obtained area against injected concentrations of nine data points, i.e., 0.0005, 0.001, 0.0025, 0.005, 0.01, 0.025, 0.05, 0.1, and 0.25 mg/L. The lowest calibration level (LCL), 0.0005 mg/L, accounts for a sample of 0.005 mg/kg (half of the LOQ), and 0.25 mg/L represents a 2.5 mg/kg sample. R^2^ values are listed in Table 1 and were all very close to the “1” value, showing that the change (increment) in response (area) is similar to the change in concentration across the selected points’ range; hence, high-quality sample measurement was achieved. The matrix effect was minor and mainly suppressive (Table 1). The cucumber matrix was the most effective, with an average of −9.8% for all compounds and −20.9% for dinotefuran. Tomato had the most minor matrix effect. According to the criteria of the European Union, matrix effects within the range of −20% < ME < 20% are considered negligible. Therefore, insignificant matrix effects were observed for the analytes tested in the tomato, cucumber, and lettuce samples, except dinotefuran in cucumber, which had a moderate effect. The 0.01 mg/kg as the lowest MRL was set as the LOQ and achieved a satisfactory recovery and relative standard deviation (RSD) of 92.2–113% and <8.9%, respectively, of all tested matrices (Table 2). The precision of the method was assessed in the three matrices at the LOQ level of 0.01 mg/kg in terms of intra-day repeatability (pooled RSDr), which was less than 6.1%, and inter-day repeatability (pooled RSD_R_), which was less than 6.9% (Table 1), which matches the European Union criteria for precision (≤20%) and recovery (70–120%) [29]. The percentages of recovery were between 91.9% and 114.4% in all matrices at the three spiking levels (Table 2), which is acceptable by the European Union criteria (70–120%) [29].

### 3.2. A Comparison of the Developed Method with the Previous Studies

The developed method is superior to the previous methods [30,31,32,33,34,35,36,37] in terms of the recovery rates, time required for complete extraction, solvent consumption, and operating efficiency (Table S2). Our developed method’s recovery percentage of the seven target neonicotinoids is sufficiently high. Although the LOQs of the target analytes are not lower than those of the previously published methods [28,29,30,31,32,33,34,35], they are still well below the established MRLs. Regarding organic solvent consumption, no organic solvents are required for extraction and solvent exchange compared to the high solvent consumption of the previous methods [30,31,32,33,34,35,36,37]. In addition, some methods used an SPE cartridge for the cleanup step [30,36,38], whereas our proposed method does not require additional purification steps. Reducing the analysis time was an essential focus of our research. It was found that the extraction time in this study was 1 min, so this method is more environmentally friendly, economical, and rapid.

The AGREE metric approach, developed by Pena-Pereira et al. (2020), is used to evaluate the environmental greenness of an analytical method [39]. The metric process compares analytical methods based on the 12 principles of green chemistry. Each principle is labeled in a pictogram and scored from 0 to 1. The AGREE Metrics software version 0.5 beta [23] requests data input for the twelve assessment principles. After scoring, a circular pictogram with a circle in the center displays the total score and the segments corresponding to the twelve criteria, each with a color corresponding to the assigned weight. The color of each element changes after the assessment and then indicates the process’s strengths and weaknesses and their contribution to the overall score.

The method proposed by Watanabe et al. (2015) [36] was compared with the method developed in this study. Both procedures are based on water extraction to allow for a fair comparison. A weight of 2 was set for all 12 principles evaluated (assuming that all evaluation criteria are equally important). For Principle 1 (sample preparation), the compared methods were offline analyzed (in the laboratory), and a score of 0.48 was obtained. For Principle 2 (sample weight), the proposed method used a sample weight of 3 g (0.49 score), while the Watanabe et al. method used 5 g (0.42 score). Since the location of the analytical equipment (Principle 3) for both compared methods must be in the laboratory, both methods scored 0. The proposed method outperforms the method of Watanabe et al. in terms of the number of main steps for sample preparation (Principle 4). The proposed method includes only two main steps: sample extraction and LC-MS/MS analysis (score 1), which were fewer than the method by Watanabe et al. (score 0). The method steps were performed manually, were not miniaturized (Principle 5) for both compared methods, and achieved a score of 0. The compared methods do not contain derivatization steps (Principle 6) and achieve similar scores of 1. A major advantage of the proposed method over the Watanabe et al. method is the amount of analytical waste it generates (Principle 7). The method by Watanabe et al. scored 0.1 due to the high consumption of solvents and adsorbents to purify the extract, whereas the proposed method scored 0.21. There are seven analytes analyzed per run in both methods. In contrast, the sample throughput per hour is 2 for Watanabe et al. and 4 for the method proposed in this study (Principle 8), achieving a score of 0.59 and 0.76, respectively. The chromatography and laboratory equipment used for the determination are energy-intensive. Watanabe et al.’s method used the LC-UV chromatographic technique (0.5 scores), whereas the proposed developed method used LC-MS/MS (0 scores) (Principle 9). Therefore, Principles 3, 5, and 9 did not correspond to analytical greenness and received the same score of zero. The proposed developed method used water for extraction (score 1), whereas the reagents and solvents in the method by Watanabe et al. were not from bio-based sources (score 0) (Principle 10). The Watanabe et al. method used large volumes of acetonitrile, methanol, and toluene for sample preparation and purification, considered toxic solvents (Principle 11), and achieved a score of 0.03. In contrast, the proposed method used only a small amount of methanol (1.92 mL) in the mobile phase for chromatographic separation and received a score of 0.42. The aquatic life hazards, bioaccumulation, persistence, high flammability, explosiveness, and corrosiveness of the solvents used in the Watanabe et al. method cannot be avoided (0 scores) (Principle 12). In contrast, the proposed developed method achieved a score of 1.

**Figure 1:**
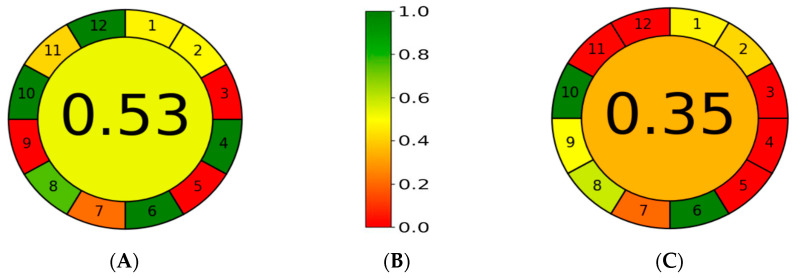
A comparison of the greenness principles of the proposed developed method (**A**) in this study and Watanabe et al.’s method [26] (**B**) and the corresponding color scale for reference (**C**).

The overall analysis of the AGREE results showed that the proposed method had an overall score of 0.53 compared to the method by Watanabe et al., which received a score of 0.35, indicating that the proposed method is greener than the reported method (Figure 1).

### 3.3. Real Samples

To ensure the efficiency of the developed method, real marketed samples were analyzed for comparison purposes using the developed method in this study and the standard EN QuEChERS method [24]. Data obtained from five positive samples for each matrix are shown in Table 3. Only four compounds were detected, i.e., thiamethoxam, imidacloprid, acetamiprid, and thiacloprid. Dinotefuran and nitenpyram were not detected in any sample. Thiacloprid was detected in one tomato sample only, thiamethoxam was found in one lettuce sample and two cucumber samples, and imidacloprid (7 samples) and acetamiprid (6 samples) in all matrices. The *t*-test was performed to test the residue data obtained by the developed method in this study and the standard EN QuEChERS method with a 95% confidence level. The statistical analysis results showed no significant differences between the data obtained by both methods; the *p*-value was above 0.05 (*p* ≥ 1.51), ensuring the developed method’s efficiency.

### 3.4. Dissipation of Imidacloprid and Dinotefuran

Imidacloprid and dinotefuran are registered to control white fly Bemisia tabaci in tomato fields. Comparable application rates of active ingredients in spray solution are recommended (21 g of imidacloprid and 25 g of dinotefuran). Comparable initial deposits of both compounds in tomato fruits were observed: 0.75 mg/kg for imidacloprid and 1.18 for dinotefuran. The residues of imidacloprid and dinotefuran found in tomatoes against time are presented in Figure S2.

Using Microsoft Excel, the dissipation rate was calculated by plotting the natural logarithm (ln) of the found residues (mg/kg) against time in days. The dissipation rate k = the slope of the regression line. The regression line equation was utilized to calculate T_½_ and PHI values. Dissipation followed first-order kinetics in both compounds. The R^2^ values were 0.9561 and 0.9689 for imidacloprid and dinotefuran, respectively, showing a good fit of the obtained field residue data with the regression line. Dinotefuran showed a faster degradation rate K (0.2781) and a shorter T_½_ value of 2.5 days than in the case of imidacloprid (k = 0.2013 and T_½_ = 3.4 days) (Table 4).

Many factors affect pesticide dissipation, including the physical and chemical properties of the compound, such as vapor pressure and water solubility, and plant-specific characteristics, such as the growth rate, plant morphology, and microbial activity, in addition to environmental factors, such as rainfall, temperature, and the duration of direct sunlight [18,40,41,42,43]. The Octanol-Water partition coefficients (log P) of imidacloprid and dinotefuran are 0.57 and −0.549, respectively [44], suggesting that imidacloprid is more hydrophobic than dinotefuran. Dinotefuran was also found to volatilize faster than imidacloprid, which can be explained by the high vapor pressure of dinotefuran (0.0017 mPa at 20 °C) compared to imidacloprid (4 × 10^−7^ mPa at 20 °C) [44]. The results clearly show the difference in the initial deposition of the tested neonicotinoids, as the initial deposit of dinotefuran was higher than that of imidacloprid. The applied dose was higher for dinotefuran than imidacloprid, which could explain this difference. Another reason is the different types of formulation, which could also play an essential role in the dissipation of the pesticides. In this study, it is hypothesized that the observed significant difference between the half-lives of dinotefuran and imidacloprid in tomato fruit may not be due to the effect of dilution during plant growth, as both pesticides were applied at the same time and the same stage of fruit formation [45] and without rainfall.

The pre-harvest interval (PHI) is influenced, among other factors, by the MRL value. According to EU legislation, the MRL value of imidacloprid is 0.3 mg/kg and dinotefuran is 0.01 mg/kg. Although the rate of the dissipation of dinotefuran was faster than that of imidacloprid, as shown by T_½_ and k values, the PHI of dinotefuran in tomatoes is longer (about twice as long) than that of imidacloprid. This is due to the lower MRL of dinotefuran, which is the detection limit in the European Union. Meanwhile, for Codex regulations, the MRL value of both imidacloprid and dinotefuran is 0.5 mg/kg [46]. According to MRLs of Codex, the estimated PHI is three days for both compounds (Table 4). The dissipation of imidacloprid was studied in different crops. The half-life value was 6.5 to 7.2 days in Chinese Prickly [47]; in another study, T_½_ was 8 to 11.1 days in pomegranate (54 to 108 g a.i./ha), and PHI was 1 day in all cases [48]. The residues of imidacloprid were lower than the MRL in green beans and chili peppers treated by adding imidacloprid to irrigation water (fertigation); hence, no PHI was required [49]. These differences may be partly due to the plant variety, application doses, local environment, and differences in plant growth.

### 3.5. Risk Assessment of Residues of Field Trial

The estimation of chronic and acute risks was performed based on the highest acquired residue data from the field trial for imidacloprid (0.75 mg/kg) and dinotefuran (1.18 mg/kg) (Table 4). The maximum residues found (residues at zero days after application) were used as the worst-case scenario.

The chronic risk was estimated by comparing real intake against the set acceptable daily intake (ADI). The ADI of imidacloprid and dinotefuran is 0.06 and 0.2 mg/kg body weight per day, respectively [43], thus 3.6 and 12 mg per day per person of 60 kg [50]. The estimated daily intake (EDI) of imidacloprid and dinotefuran via tomato (44.1 g of average consumption for Mediterranean citizens) was calculated to be 0.03 and 0.05 mg for imidacloprid and dinotefuran, respectively, at the maximum found residues level per person. Tomato consumption (44.1 g) represents only 3.3% of the total consumed food per day (1342.5 g) per person in the Middle East, meaning that the allowed daily intake of imidacloprid and dinotefuran via tomato should not exceed 3.3% of the ADI for each compound. Consequently, the maximum allowed intake from tomatoes is 0.12 and 0.4 mg per person daily for imidacloprid and dinotefuran, respectively. Accordingly, the chronic risk factor was estimated to be 25% and 13% of the allowed amount taken from tomatoes, indicating a low risk of both compounds, particularly for dinotefuran.

The Acute Reference Dose (ARfD) was considered the criterion for estimating acute risk. ARfD values are 4.8 mg per person for imidacloprid [51] and 75 mg per person for dinotefuran [52]. The acute risk factor was rounded to 0.63% and 0.07% for imidacloprid and dinotefuran, respectively. This indicates a very low acute risk in a single meal. Risk assessment data and calculations are shown in Table 5.

By studying the obtained data from field trials and risk estimation, it can be concluded that dinotefuran showed a faster dissipation rate than imidacloprid. In addition, dinotefuran has a much lower risk from a chronic and acute perspective than imidacloprid. The MRL for dinotefuran set by the EU could restrict the use of dinotefuran for manufacturers wishing to offer their products to the European market. The EU-MRL value for dinotefuran should be reconsidered, as it results in a longer PHI, to allow for alternative neonicotinoids to control white flies and other insect pests and suppress potential resistance [53].

## 4. Conclusions

A rapid, cheap, effective, and environmentally friendly method based on vortex-assisted liquid extraction using only water as green extraction solvent without salting out and cleanup steps and using ultra-performance liquid chromatography/tandem mass spectrometry (UPLC-MS/MS) was optimized for the simultaneous determination of seven neonicotinoids in fresh vegetables including tomato, cucumber, and lettuce samples. Various parameters of the method were tested, and the best combination was selected. The developed method was validated according to the European Union guidelines. The accuracy, precision, matrix effect, and LOQ were assessed, and the obtained values were accepted according to EU criteria. The advantages of the proposed method include high extraction efficiency, low matrix effects, no organic toxic solvents required for extraction, and time-saving. The extraction method can be considered environmentally friendly, safe, and straightforward. It could be an excellent alternative to the techniques currently used to analyze neonicotinoids in vegetables and fruits of high water content at sufficient sensitivity and low concentrations below the EU’s MRLs. In addition, real marketed samples were analyzed using the developed method. The results were compared with those obtained using the standard EN QuEChERS method [22] for the same samples, and no significant differences were noted. The dissipation behavior of imidacloprid and dinotefuran was investigated in tomatoes grown in open fields, and the half-life and PHI were calculated. Chronic and acute risk factors were assessed using data from the field trial. Dinotefuran had lower risk factors than imidacloprid. However, the levels of residues of both compounds were safe for consumers.

## Figures and Tables

**Table 1:** The linearity (R^2^), matrix effect (%), LOQ (mg/kg), and precision of the method.

	R^2^			Matrix Effect %			LOQ ^a^ (mg/kg)	Precision at 0.01 mg/kg		
	Tomato	Lettuce	Cucumber	Tomato	Lettuce	Cucumber		%R ^b^	RSD_r_^c^	RSD_R_^d^
Dinotefuran	0.9999	0.9999	0.9999	−10.8	−15.8	−20.9	0.01	103.0	6.1	6.9
Nitenpyram	0.9997	0.9998	0.9998	10.5	−1.5	−5.6	0.01	102.2	5.2	5.8
Thiamethoxam	0.9997	0.9998	0.9998	−0.2	−1.6	−4.3	0.01	99.8	5.3	2.6
Clothianidin	0.9996	0.9997	0.9998	−0.6	0.4	−0.8	0.01	106.8	5.1	6.4
Imidacloprid	0.9999	0.9999	0.9999	−1.8	3.2	−1.7	0.01	103.2	5.7	3.1
Acetamiprid	0.9999	0.9999	0.9998	−11.8	−16.8	−19.2	0.01	100.4	4.4	4.0
Thiacloprid	0.9998	0.9999	0.9999	−12.2	−13.7	−15.9	0.01	101.4	4.8	4.6

^a^ LOQ was set at lowest MRL value according to EU legislation. ^b^ mean recovery percentage of tested analytes (*n* = 5, each matrix). ^c^ intra-days repeatability (pooled RSDr) (*n* = 5, each matrix). ^d^ Inter-days repeatability (pooled RSD_R_) (*n* = 15, each matrix, 3 different days, 7 day intervals).

**Table 2:** Recovery (%) (RSD%) of analyzed neonicotinoids at three concentration levels in tomato, lettuce, and cucumber samples.

	Tomato			Lettuce			Cucumber		
	Spiking Levels (mg/kg)			Spiking Levels (mg/kg)			Spiking Levels (mg/kg)		
	0.01	0.1	1	0.01	0.1	1	0.01	0.1	1
Dinotefuran	107.1 (7.5)	107.0 (2.3)	103.0(1.1)	106.0 (4.8)	102.6 (7.5)	113.2 (13.1)	101.7 (8.3)	91.9 (4.7)	94.8 (5.3)
Nitenpyram	104.6 (6.1)	108.8 (2.7)	102.1(1.3)	106.2 (8.9)	94.9 (5.5)	107.0 (10.2)	92.2 (4.6)	109.7 (5.1)	94.5 (2.7)
Thiamethoxam	97.5 (1.2)	102.1(0.4)	100.9(2.3)	99.5 (5.2)	108.3 (11.4)	101.9 (12.9)	97.5 (2.3)	94.1 (4.6)	96.4 (7.6)
Clothianidin	113.0 (3.2)	101.3 (1.8)	107.6(4.1)	104.0 (8.1)	109.6 (13.5)	105.8 (0.4)	97.9 (3.6)	113.2 (4.7)	108.5 (6.2)
Imidacloprid	102.6 (5.1)	114.4 (2.9)	101.3(1.9)	101.9 (4.7)	109.5 (9.6)	105.0 (14.6)	93.4 (1.5)	100.1 (5.7)	100.3 (5.6)
Acetamiprid	103.7 (3.8)	104.7 (4.1)	102.2(1.3)	98.3 (5.4)	107.2 (7.4)	98.7 (5.3)	94.5 (4.0)	93.8 (3.0)	100.3 (5.3)
Thiacloprid	103.3 (2.7)	105.0 (1.2)	100.3(0.4)	113.7 (8.1)	107.8 (10.7)	103.7 (9.1)	92.8 (1.5)	92.0 (5.5)	93.7 (3.9)

**Table 3:** Residues found (mg/kg) in the positive marketed samples analyzed using the developed method (DM) and QuEChERS EN (Q EN) method [22].

Samples	No.	Thiamethoxam		Imidacloprid		Acetamiprid		Thiacloprid	
		DM	Q EN	DM	Q EN	DM	Q EN	DM	Q EN
Tomato	1	-	-	-	-	0.24	0.28	-	-
	2	-	-	0.29	0.24	-	-	-	-
	3	-	-	0.66	0.69	-	-	-	-
	4	-	-	-	-	-	-	0.12	0.11
	5	-	-	-	-	0.13	0.11	-	-
Lettuce	1	-	-	0.81	0.77	-	-	-	-
	2	-	-	0.39	0.42	-	-	-	-
	3	-	-	-	-	0.12	0.15	-	-
	4	0.71	0.63	-	-	1.34	1.11	-	-
	5	-	-	-	-	0.13	0.12	-	-
Cucumber	1			0.69	0.77	0.52	0.43	-	-
	2	0.30	0.22	-	-	-	-	-	-
	3	-	-	0.93	0.84	-	-	-	-
	4	-	-	0.44	0.49	-	-	-	-
	5	0.15	0.13	-	-	-	-	-	-

**Table 4:** Dissipation parameters and PHI of imidacloprid and dinotefuran in open field-grown tomato.

	Imidacloprid	Dinotefuran
Intercept ^a^	−0.1733	−0.014
Slope ^b^	−0.2013	−0.2781
K	0.2013	0.2781
R^2 c^	0.9561	0.9689
T_½_^d^	3.4 days	2.5 days
PHI, EU	7 days	14 days
PHI, Codex	3 days	3 days

^a,b^ intercept and slope of the semi-logarithmic graph, respectively. ^c^ determination coefficient. ^d^ T_½_ = ln (2)/k.

**Table 5:** Estimated chronic and acute risk assessment of imidacloprid and dinotefuran after consumption of open-field tomatoes.

	Imidacloprid	Dinotefuran
ADI/person ^a^, mg	3.6	12
Tomato consumption/day/person, g [50]	44.1	44.1
Total food consumption/day/person, g [50]	1342.5	1342.5
Tomato percentage of total food consumption ^b^	3.3%	3.3%
Maximum found residues, mg/kg ^c^	0.75	1.18
EDI via tomato per person, mg ^d^	0.03	0.05
Maximum allowed daily intake of tomato per person, mg ^e^	0.12	0.4
Chronic risk factor % ^f^	25	13
ARfD per person, mg	4.8 ^g^	75 ^h^
Acute risk factor % ^i^	0.63	0.07

^a^ according to Codex Alimentarius and calculated for average person of 60 kg. ^b^ tomato consumption÷total consumption×100=3.3%. ^c^ initial deposits from field trial. ^d^ EDI=residues concentration÷1000×44.1. ^e^ ADI per person×3.3÷100. ^f^ CRF=EDI per person÷ADI per person×100. ^g^ according to EU database and calculated for average person of 60 kg. ^h^ according to EPA. ^i^ ARF=EDI per person÷ARfD per person×100.

## Data Availability

The original contributions presented in the study are included in the article/Supplementary Material, further inquiries can be directed to the corresponding authors.

## Supplementary Materials

The following supporting information can be downloaded at https://www.mdpi.com/article/10.3390/foods13071106/s1, Figure S1: Representative chromatograms of the 7 separated neonicotinoids at a concentration of 10 µg/L in DI water using LC-MS/MS, Figure S2: Residues of imidacloprid and dinotefuran in tomatoes under field conditions. Table S1: LC–MS/MS parameters for determination of neonicotinoids. Table S2: A comparison of the extraction/cleanup step, recovery, and LOQ of neonicotinoids in the previous studies with the current study.
