# Supplementary material for: Development of Green and Facile Sample Preparation Method for Determination of Seven Neonicotinoids in Fresh Vegetables, and Dissipation and Risk Assessment of Imidacloprid and Dinotefuran
Source: Foods. 2024 Apr 4;13(7):1106. doi: 10.3390/foods13071106 (PMC11011385; doi:10.3390/foods13071106)
Supplement: Supplementary file 1 [file foods-13-01106-s001.zip › foods-2902424-supplementary.pdf]

Supporting Information

Development of green and facile sample preparation method for the determination of seven neonicotinoids in fresh vegetables, and dissipation and risk assessment of imidacloprid and dinotefuran

Osama I Abdallah[1*], Rania M. Abd El-Hamid[1], Nevein S. Ahmed[1], Saleh S. Alhewairini[2]. Sherif B. Abdel Ghani[2, 3*]

[1] Department of Pesticide Residues and Environmental Pollution, Central Agricultural Pesticide Laboratory, Agricultural Research Center, Giza, 12618, Egypt.

[2] Department of Plant Protection, College of Agriculture and Food, Qassim University, P.O. Box 6622, Buraydah, Saudi Arabia.

[3] Department of Plant Protection, Faculty of Agriculture, Ain Shams University, P.O. Box 68 Hadayek Shoubra, 11241 Cairo, Egypt.

[*] Corresponding author.

E-mail addresses: oiabdullah@qassim.gov.sa (O. I. A.), sherifbiomy@yahoo.com (S. B. A.)

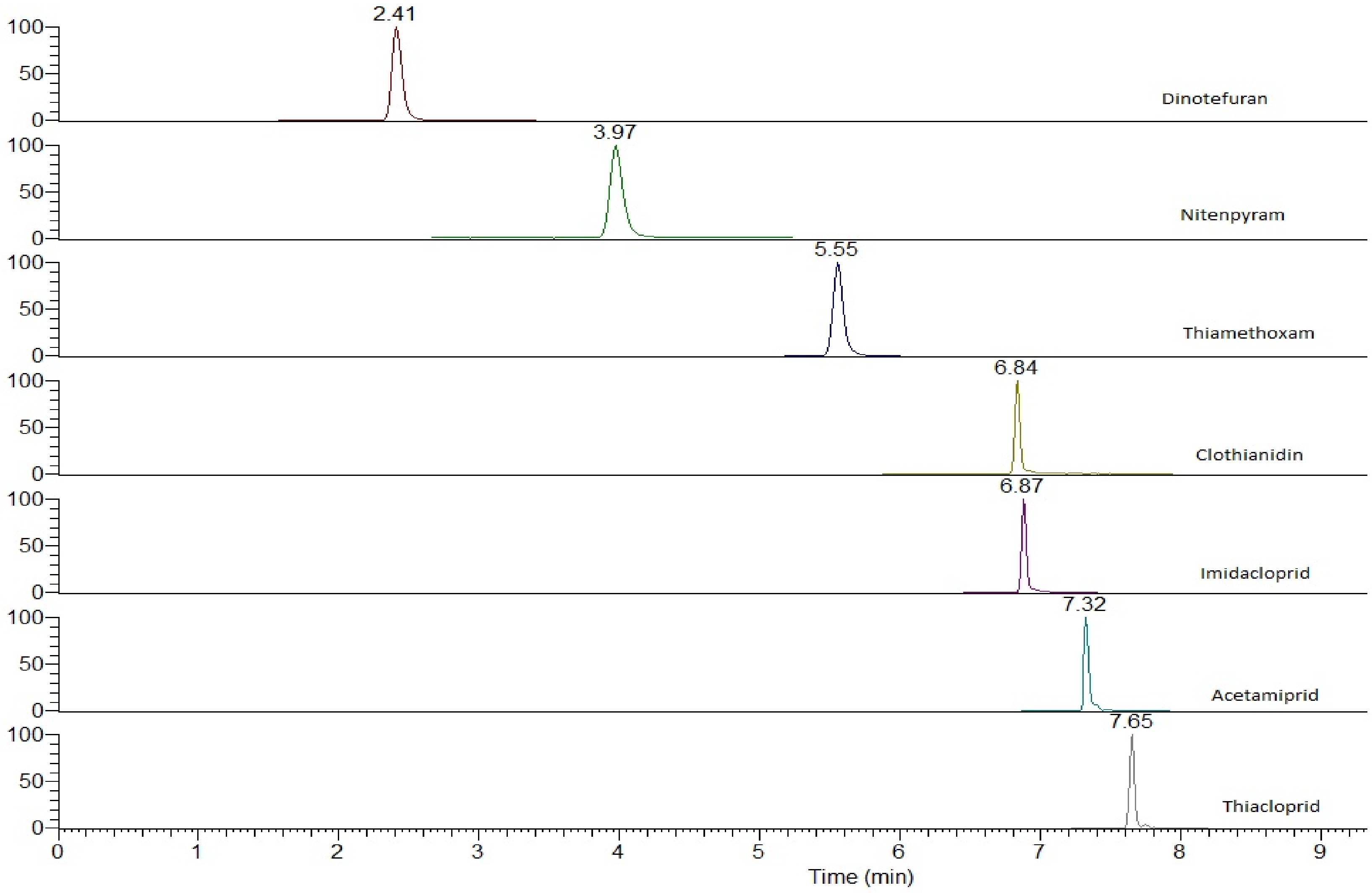
2.41
Dinotefuran
3.97
Nitenpyram
5.55
Thiamethoxam
6.84
Clothianidin
6.87
Imidacloprid
7.32
Acetamiprid
7.65
Thiacloprid
100
50
0
0
1
2
3
4
5
6
7
8
9
Time (min)

Figure S1. Representative chromatograms of the 7 separated neonicotinoids at concentration of 0.01 μg/L in DI water using LC-MS/MS

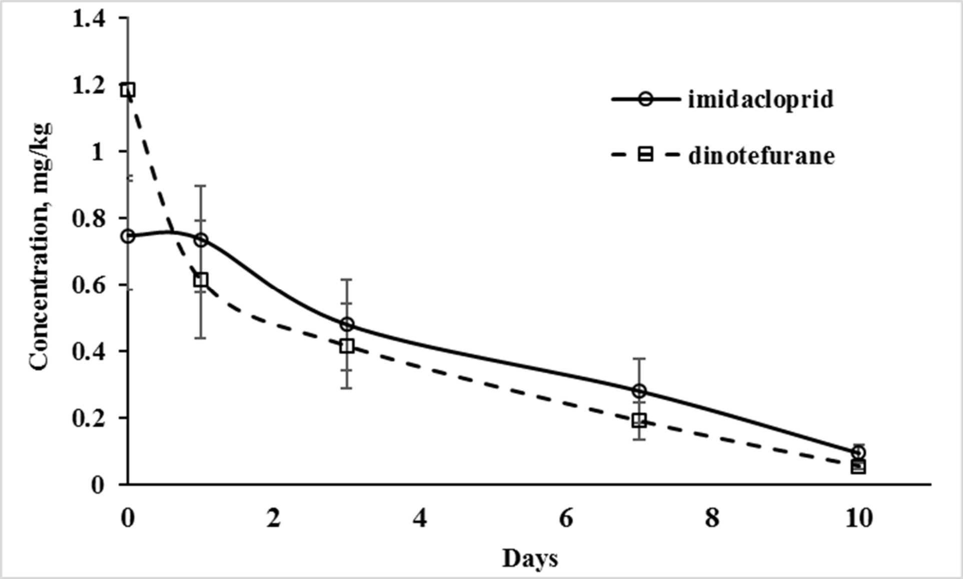

Figure S2. Residues of imidacloprid and dinotefuran in tomatoes under field conditions.

Table S1. LC–MS/MS parameters for determination of neonicotinoids

	Retention Time (min)	Precursor (m/z)	Product (m/z)	Collision Energy (V)	RF Lens (V)
Dinotefuran	2.51	203	113.1	10.2	36
			**129.05**[a]	12.4	36
Nitenpyram	4.11	271.06	189.1	14.2	50
			**225.1**	12.09	50
Thiamethoxam	5.67	292.08	181.08	23.7	40
			**211.1**	12.3	40
Clothianidin	6.88	250.03	131.9	17.09	46
			**169.05**	13.2	46
Imidacloprid	6.91	256.1	175.08	20.08	50
			**209.1**	16.7	50
Acetamiprid	7.34	223.03	90.07	34.7	52
			**125.9**	21.6	52
Thiacloprid	7.66	253	99.04	42.2	59
			**126.04**	21.8	59

[a] Product ions in bold were utilized for quantification

Table S2. Comparison of the extraction/clean-up step, recovery, and LOQ of neonicotinoids in the previous studies with the current study

No of analytes	Matrices	Instrument	Extraction/cleanup	% Recovery	LOQ (µg/kg)	Ref.
5	Vegetables and fruits	LC-MS (APCI)	-Sample weight: 20 g<br>-Extraction solvents: 95 mL methanol<br>-Clean-up: SPE cartridge	70-95	10 (20 for nitenpyram)	[30]
5	Apple	LC-MS/MS (APCI)	-Sample weight: 10 g<br>-Extraction solvents: 10 mL acetonitrile, followed by salting out step.<br>-Clean-up: dSPE using primary secondary amine (PSA)	93-108	2-3	[33]
3	Cabbage, Tomatoes, Chilli, Peppers, and Potatoes	HPLC-UV	-Sample weight: 1 g<br>-Extraction solvents: Microwave Assisted Extraction (MAE), acetone, n-hexane, and CH_2CL_2	68.1-106	3.2-4.8	[32]
4	Tomatoes Cucumber	HPLC-UV	-Sample weight: 10 g (DSPE)<br>-Extraction solvents: 10 mL acetonitrile, followed by salting out step.<br>-Clean-up: dSPE using PSA and MWCNTs, followed by DLLME<br>-Procedure for enrichment using $CHCL_3$	84.6-97.5	0.5-1	[34]
6	Spinach, Cucumber, Apple, and Pomelo	LC-MS/MS	-Sample weight: 10 g<br>-Extraction solvents: 10 mL acetonitrile, then salting out<br>-Clean-up: dSPE using primary secondary amine (PSA) and graphitized carbon black (GCB)	73.7-103.8	0.66-2.84	[35]
4	cereals, vegetables, fruits	UPLC-MS/MS	-Sample weight: 10 g<br>-Extraction solvents: 5 mL water and 10 mL acetonitrile, followed by salting out step.<br>-Clean-up: dSPE using primary secondary amine (PSA) and graphitized carbon black (GCB)	82.7-103.4	0.71-2.26	[31]
7	Cucumber, Eggplant	HPLC-UV	-Sample weight: 5 g<br>-Extraction solvents: 25 mL water<br>-Clean-up: SPE, Oasis HLB, and Envi-Carb cartridges	82-114	7-17,<br>3-19	[36]
4	Diverse crops	HPLC-UV	-Sample weight: 10 g<br>-Extraction solvents: 5 mL water and 5 mL acetonitrile, followed by salting out step.<br>-Clean-up: dSPE using PSA and GCB.	77.5-96.4	0.2 µg/mL	[37]
7	Tomatoes Cucumber Lettuce	UPLC-MS/MS	-Sample weight: 3 g<br>-Extraction solvents: 27 mL water<br>-No cleanup step.	91.9-113.7	10	This study
